# Uniform pore morphology of bijel-templated materials reduces cell circularity and inflammatory expression of macrophages

**DOI:** 10.1016/j.mtbio.2026.103175

**Published:** 2026-04-29

**Authors:** Alyse R. Gonthier, Elliot L. Botvinick, Ali Mohraz

**Affiliations:** a Department of Materials Science and Engineering, University of California, Irvine, CA, 92697, USA; b Department of Biomedical Engineering, University of California, Irvine, CA, 92697, USA; c Department of Surgery, University of California, Irvine, CA, 92697, USA; d Beckman Laser Institute, University of California, Irvine, CA, 92697, USA; e Edwards Lifesciences Foundation Cardiovascular Innovation and Research Center, University of California, Irvine, CA, 92697, USA; f Department of Chemical and Biomolecular Engineering, University of California, Irvine, CA, 92697, USA

**Keywords:** Bijel, Macrophage, Inflammation, Porous material, Cell shape

## Abstract

The design of implantable biomaterials often aims to guide local cell behavior to control immune response. Non-chemical routes exploit the effect of mechanical properties and structure morphology on cell behavior. A class of substrates known as bicontinuous interfacially jammed emulsion gel (bijel)-templated materials (BTMs) exhibit characteristically uniform pore size and surface curvature. Existing research on these materials in vivo demonstrates their ability to modulate the inflammatory response in the anti-inflammatory direction. We investigate a subset of the contributing components to that effect by examining the behavior and phenotype of macrophages within BTMs in vitro. The comparative substrate, the particle-templated material (PTM), has an equivalent chemical composition, but has variable pore size and surface curvature. Macrophages within these two materials take on notably different cell shapes and phenotypes. Macrophages interacting with the BTM exhibit less circular cell shapes and a lower state of inflammation. This effect is significant enough to induce lower pro-fibrotic activation in fibroblasts, without direct BTM-fibroblast contact. These results suggest that microscale curvature and pore size have a direct effect on macrophages, and that this effect can cause phenotypic changes in other cells. Findings reaffirm the significance of targeting macrophages in biomaterials design and support further investigation of the immune signaling cascade that occurs within BTMs. Our contributions to the fundamental knowledge of cell behaviors in these porous materials provide new insights applicable to advancing biomaterials design.

## Graphical abstract

**Figure:**
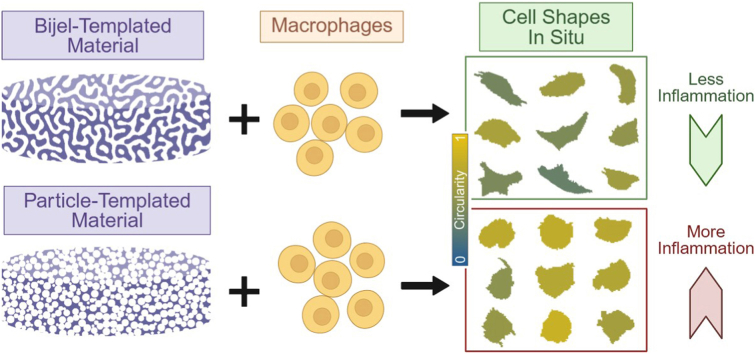

## 1 Introduction

Advancements in biomaterial design are hallmarked by the ability of those materials to dictate cell behavior [[1], [2], [3]]. Utilizing biomaterial structure alone to influence cell behavior is continually a popular focus area [[4], [5], [6]], in part due to a lack of concern for unintended side effects, in contrast to chemical manipulation [7,8]. Many studies evaluate the effect of biomaterial structure on overall immune response in vivo [9,10], but do not always continue the investigation at the fundamental level to parse the specific cell-material relationships at play. Examining how biomaterial architecture influences the behavior of individual cell types, and how that relationship is related to the overall in vivo response, provides critical data needed to inform future biomaterial development.

Previous studies investigating porous materials in vivo have demonstrated that pore size has an immune-modulating effect [11,12], while in vitro studies on other materials suggest that substrate curvature influences cell migration and phenotype [[13], [14], [15]]. The inherent relationship between these two microstructural metrics makes it challenging to investigate one with limited influence of the other. Our previous in vivo study, which examined substrates with the same pore size but different pore structures, found that the substrate with a more uniform pore size distribution and consistent surface curvature resulted in less fibrosis, increased vascularization, and beneficial response from immune cells [9]. This favorable in vivo result warrants a detailed investigation to develop a clearer understanding of how the structural characteristics of that material affect key cell types implicated in fibrosis, vascularization, or immune response; it is not known which are directly affected by the pore structure and which may be affected downstream by cell-cell signaling.

**Fig. 1:**
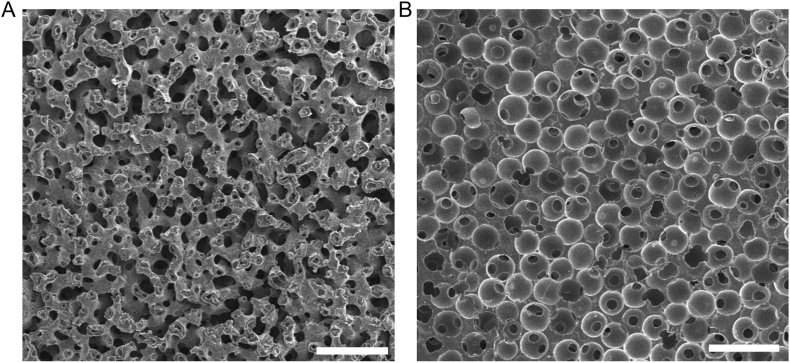
Porous substrates visualized via scanning electron microscopy. Poly(ethylene glycol) diacrylate samples of the BTM (A) and PTM (B) are pictured. Scale bar, 100 μm.

The promising material described is known as a BTM, or bicontinuous interfacially jammed emulsion gel (bijel)-templated material [16,17]. These materials have a fully continuous and relatively uniform pore structure with negative Gaussian (saddle-like) curvature [18] along their internal surfaces, characteristic of the bijel from which they are derived (Fig. 1A). Creating a BTM starts with making a bijel, which forms by the spinodal decomposition (spontaneous demixing) of two partially miscible fluids in the presence of neutrally-wetting particles, which sequester at the fluid-fluid interface during demixing [[19], [20], [21]]. Once the interfacial area is reduced (by demixing) to accommodate a monolayer of such particles, the system undergoes an interfacial jamming transition, and demixing comes to a halt. Therefore, the characteristic domain size of the bijel, which in turn determines the pore size of its BTM derivative, can be kinetically controlled by the volume fraction of the neutrally wetting particles [22]. Once the bijel is formed, a monomer solution which selectively partitions into one of the fluid phases is added and photopolymerized. This results in the BTM, a porous substrate that retains the distinctive traits of the original bijel template.

When implanted in rodents, BTM samples had more pro-healing immune cell behavior in comparison to a material made from the same polymer, but with non-uniform pore sizes and variable surface curvature [9]. The latter is called a particle-templated material (PTM), which is formed from a fused volume of spherical particles. Specifically, randomly close-packed polymeric spheres are superficially fused together at elevated temperatures. The space around the spheres is subsequently polymerized (with a different chemistry) and the particles are chemically etched out, inverting the original structure to form the final PTM (Fig. 1B) [10,23]. While the spheres matched the pore size of the BTM, the sphere-connecting pore throats are generally about 1/3 the width of the sphere diameter [10,24]. A recent computational study by our group suggests that the curvature landscapes created by these two substrates induce differences in cell shape and migration behavior [25]. This provides one clue to the overall immune question, as existing research demonstrates a cause-and-effect relationship between shape and phenotype for some cell types. For example, macrophages have been shown to modulate their immune state based on elongation, where an elongated macrophage tends to be pro-healing (M2) as opposed to pro-inflammatory (M1) [26]. Another study demonstrated that cells with a less spherical induced shape also produce less inflammatory cytokines [27]. Macrophages are especially useful in initial investigations of biomaterials’ impact on cell behavior, thanks to their well-established activation paradigm, where the classically activated (M1) versus alternatively activated (M2) macrophages have known phenotype expressions [28,29]. While further research has shown additional sub-phenotypes and other nuanced behaviors [30], the general behaviors and associations of the M1 and M2 activation states are widely accepted and utilized [31,32]. This classification provides a ubiquitous benchmark for examination of the immunomodulatory effects of biomaterials. Another cell of interest is the fibroblast, which is a critical player in the foreign body response and is known to communicate with macrophages [33]. Fibroblasts are builders of the extracellular matrix at the wound site, and thus the primary contributors to fibrosis leading to encapsulation [34]. However, fibroblasts do not have the same well-established inflammatory phenotypes as macrophages and are often measured for their inflammatory contributions by proxy, via factors associated with the myofibroblast transition [35], without a pro-healing counterpart. Some studies describe a potential connection between fibroblast shape or elongation and fibrotic activation, but these relationships have not been widely replicated [36]. Fibroblasts and macrophages, then, represent an intriguing pairing for studying the effects of microstructural cues on cell behavior, particularly relevant to foreign body response to implanted biomaterials.

The differences in pore landscape correlated to in vivo immune response, in combination with existing relationships between shape and phenotype, motivate a specific investigation of the connection between immune cell behavior and physical cell traits within the BTM and PTM. In this paper, we examine the behavior of macrophages and fibroblasts seeded into these substrates, quantifying the cell shape, size, and phenotype. This study also seeks to understand how one cell-substrate interaction may subsequently affect another. To that end, a conditioned media study is performed by analyzing the phenotype of fibroblasts introduced to the substrate-macrophage chemical environment. Collectively, the results presented herein provide new insights into the immunomodulatory capability of morphologically distinct porous biomaterials.

## 2 Materials and methods

### 2.1 Preparation of substrates

#### 2.1.1 Bijel-templated material (BTM)

BTMs were made according to previously published procedures [9,16]. Briefly, fluorescent silica nanoparticles were synthesized, using the Stöber synthesis method [37], by overnight mixing Rhodamine B (Sigma-Aldrich) conjugated (3-aminopropyl) triethoxysilane (APTES, TCI America), tetraethyl orthosilicate (TEOS, Sigma-Aldrich), ethanol, and strong ammonium hydroxide solution (Fisher Chemical). The particles were then washed by centrifugation with deionized water three times (VWR Clinical 200, 2500 rpm, 12 min) and dried in a vacuum oven at approximately 135 °C. The drying process results in particles that are neutrally wetting between a water-rich phase and 2,6-lutidine-rich phase, such that particles in a binary mixture of the two will rest at their interface [38,39]. In our protocol, bijels were formed by first ultrasonically dispersing (Branson Sonifier 250, Emerson) these particles in water (1.5% v/v) and mixing the appropriate amount of the dispersion with 2,6-lutidine (mole fraction x_L_ = 0.064). This mixture was pipetted into a glass, cylindrical (5 mm diameter) vessel, and quickly heated via microwave for 30 s to initiate spinodal decomposition. The vessel was capped with aluminum foil to prevent evaporation and placed in an oven for 5 min at 70 °C. A hydrogel precursor solution of polyethylene glycol diacrylate (PEGDA, Mn: 258 g/mol, Sigma-Aldrich) and photoinitiator (Darocur© 1173, 1% v/v, Ciba Specialty Chemicals) was gently pipetted on the top surface of the bijel. The vessel was recapped and placed in the 70 °C oven for 4 h, to allow the polymeric solution to diffuse throughout the lutidine-rich phase of the bijel volume. The vessel was polymerized via UV light (100 W/cm^2^, λ = 320-390 nm) for 2 min, resulting in a solid, microporous substrate at 36.2% v/v PEGDA. The polymeric BTMs were washed in isopropyl alcohol and water to remove excess precursor solutions and cut into disks approximately 1 mm in thickness. The silica particles remaining at the solid interface were removed via overnight hydrofluoric acid (HF) soak. Substrates were copiously washed in water and then soaked in aqueous sodium persulfate solution for at least 1 h (0.2 g/mL, Sigma-Aldrich). Samples were subsequently irradiated with UV-light in 10-min intervals until optically white to degrade any residual Rhodamine B [40].

#### 2.1.2 Particle-templated material (PTM)

PTMs were made by adapting published methods [9,10]. Poly(methyl methacrylate) particles (PMMA, diameter = 32-38 μm, Cospheric) were funneled into a glass, cylindrical vessel (5 mm diameter). A 16-gauge needle was inserted into the loose mass of particles and moved in a crosshatch-like pattern, slowly moving the tip of the needle up from the bottom of the vessel. This technique prevents significant non-uniform clumping and jostles the particles into a higher-contact configuration. A syringe plunger matching the vessel diameter was then pressed firmly into the particle bed to compact it. The vessel was placed in a benchtop vacuum chamber for 5 min, to additionally ensure particle-particle contact. Samples were heated in an oven at 160 °C for 16 min and cooled for 30 min to room temperature. The exact temperature and heating time were re-established from previous methodologies to account for variations in glass transition temperature of different particle batches. The fused PMMA particles were removed from the vessel and placed in a glass petri dish prior to adding the monomer solution of PEGDA (36.2% v/v, 1% v/v Darocur© 1173) in 2,6-lutidine, with an added 0.1 mg/mL of Rhodamine B. Following photopolymerization as described in Section 2.1.1, PTM samples were cut to a thickness of approximately 1 mm and mixed in ethyl acetate (Sigma-Aldrich) overnight to remove the PMMA particles. Samples were washed twice in ethyl acetate, twice in ethanol, and twice in a 50% v/v ethanol-water mixture in preparation for soaking in aqueous sodium persulfate solution for at least 1 h (0.2 g/mL). Samples were UV-irradiated and washed three times in water. The addition and subsequent removal of Rhodamine B to the polymer solution was intended to align the chemical processing steps between the BTM and the PTM.

#### 2.1.3 Substrate preparation for cell culture

Substrates were washed 5 times in sterile 70% ethanol and soaked overnight in 70% ethanol. In a sterile cell culture hood, substrates were washed 3 times with sterile Dulbecco's phosphate-buffered saline (PBS, +MgCl_2_ +CaCl_2_, Gibco) in separate wells of a 24-well plate. Dulbecco's Modified Eagle Medium (DMEM, Gibco) was supplemented with 1% v/v penicillin-streptomycin (P/S, Gibco) and 10% v/v fetal bovine serum (Sigma-Aldrich). This solution was added to each well, mixed via pipetting, removed, and replaced. The 24-well plate containing the substrates soaking in cell culture media was placed in an incubator (5% CO_2_, 37 °C) for at least 30 min prior to cell seeding.

### 2.2 Cell culture

#### 2.2.1 Fibroblasts (normal human dermal fibroblasts, NHDFs)

Normal human dermal fibroblasts (NHDFs, adult, Lonza) were grown in T-75 flasks at 37 °C and 5% CO_2_, in supplemented cell culture media described above. Cells were grown to 70-90% confluency before passaging. Passage numbers no greater than 15 were used for all testing. Passaging was performed using 0.05% Trypsin solution on cells for 5 min while incubating. Cells were collected and centrifuged at 300g for 5 min, before resuspending in fresh media and plating (Eppendorf 5910 Ri).

#### 2.2.2 Macrophages (murine RAW 264.7 cells)

RAW 264.7 cells, a murine macrophage line (TIB-71, ATCC), were grown in T-75 flasks at 37 °C and 5% CO_2_, in supplemented cell culture media. Cells were grown to 50-70% confluency before passaging. Passage numbers no greater than 25 were used for all testing [41]. Culture media was replaced no less than every 3 days, and subcultivation ratios ranged from 1:3 to 1:5, per manufacturer recommendation. Cells were lifted with cell scrapers (ThermoFisher). The suspended cells were centrifuged at 400g for 5 min, resuspended, and plated.

#### 2.2.3 Cell viability

Both cell types were, separately, plated in glass bottom 24-well plates at a density of 100,000 cells per well (counted by hemocytometer). Transwell constructs were used in order to study the effects of any potentially leaching molecules. The constructs were carefully inserted into each well after initial cell attachment for 12 h. Prepared BTM and PTM substrates were placed individually into each transwell device, ensuring that the substrate was sufficiently submerged in culture media. After 48 h, the substrates and transwell platforms were removed. Excess culture media was discarded. A Live/Dead cell imaging kit (ThermoFisher) containing calcein AM (live, green, FITC filter) and BOBO-3 Iodide (dead, red, Texas Red filter), as well as a live nuclear stain (NucBlue, Invitrogen), were distributed to each well according to manufacturer instructions. Confocal images were taken for total viability analysis. In addition, substrate samples seeded with cells were also imaged with the Live/Dead stain as qualitative confirmation of live cell presence within the materials.

### 2.3 Cell-substrate studies

**Fig. 2:**
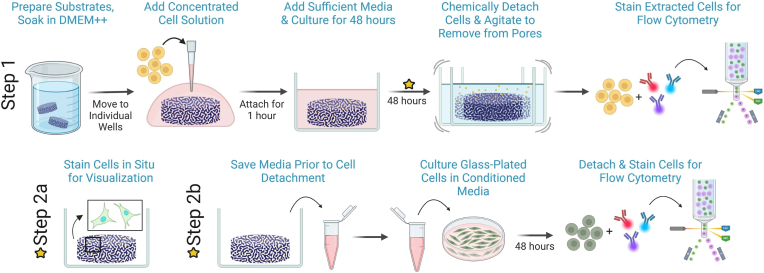
Cell-substrate studies schematic. (Step 1) Prepared substrates are hydrated in DMEM++, seeded with cells and incubated for 48 h (DMEM++ is supplemented cell culture media; see Section 2.1.3). The cells are then removed for phenotype quantification by flow cytometry. At the time point in Step 1 indicated by the yellow star, two additional options are possible. Either (Step 2a) samples are stained in situ for microscopy or (Step 2b) the media from Step 1 is reserved and used as conditioned media to culture fibroblasts for further analysis. (For interpretation of the references to color in this figure, the reader is referred to the Web version of this article.)

A flow chart of the experimental processes described below is provided (Fig. 2).

#### 2.3.1 Cell seeding in substrates

Prepared substrates were individually placed in fresh wells with minimal excess media outside of the substrate pores. NHDF and RAW 264.7 cells were lifted and centrifuged as previously described. Cells were temporarily resuspended at high concentrations of approximately 10 x 10^6^ cells/mL to minimize the total volume (8-20 μL) needed to seed ∼80,000 cells per substrate. A large volume of excess media causes a high fraction of cells to overflow onto the glass and does not allow for sufficient cell visualization in situ. A smaller overall volume of cell injection suspension was needed for RAW 264.7 cells due to their relatively smaller size. The cell suspension was injected into each substrate with a 20 μL pipette tip, pipetting small fractions of the volume at a variety of locations, with the tip resting gently on the substrate surface (Fig. 2, Step 1). The samples were incubated for 1 h to allow cells to begin attachment to the substrates. Substrates were transferred to a new 24-well plate, leaving behind any cells which were not within or attached to the substrates. An excess of media (500 μL) was then added to each well, pipetting very slowly with the pipette tip at the well wall, in order to minimize cell disturbance. Samples were incubated for 48 h before quantification or visualization.

#### 2.3.2 Fluorescent staining for microscopy

The filamentous actin (F-actin) and nuclei of both cell types were imaged via fluorescence staining (Fig. 2, Step 2a). Excess media was removed, and samples were gently rinsed with PBS. Wash steps with PBS were performed in triplicate between each of the following steps. Cells were fixed with Z-Fix (Cancer Diagnostics) for 15 min, before permeabilization with 0.1% Triton-X 100 (Thermo Scientific) for 12 min, and then blocking with 3% bovine serum albumin (BSA, ThermoFisher) for 30 min. Samples were then exposed to a solution containing AlexaFluor488 Phalloidin (1:800, ThermoFisher) and 4’,6-diamidino-2-phenylindole, dihydrochloride (DAPI, 1 μg/mL, Invitrogen) for 30 min. After final washing steps, samples were stored in an excess of PBS. Prior to imaging, samples were rotated such that the side in contact with the injection pipette was closest to the glass.

#### 2.3.3 Cell retrieval & staining for flow cytometry

Flow cytometry was performed to quantify phenotype marker expression. NHDFs were removed from the substrates via incubation with 0.05% trypsin at 37 °C and subsequent mixing by repeated pipetting of the well volume onto the substrate, in order to dislodge the fibroblasts from the substrate. RAW 264.7 cells were removed by incubating with Accutase cell detachment solution (Corning) for 1 h at room temperature and vigorous pipette mixing.

The liquid component of each sample was transferred to a microcentrifuge tube and centrifuged for 5 min at 400g. The supernatant was discarded, and the sample was resuspended in flow buffer, comprised of PBS with 1% FBS. An equal volume of Z-Fix solution was then added to each vial and samples were mixed gently. After 15 min, samples were centrifuged and resuspended in flow buffer. Each subsequent step was performed with 3 flow buffer wash steps by centrifugation. A combined permeabilization and blocking solution containing 0.1% Triton-X 100 and 3% FBS was added for 15 min. For α-smooth muscle actin (αSMA) staining of NHDF, a primary antibody solution of mouse anti-αSMA monoclonal antibody (1:200, Invitrogen) was added for 45 min, followed by a secondary antibody step containing Goat anti-mouse Cy5 (1:200, Invitrogen) and AlexaFluor488 Phalloidin (1:800). Staining of RAW 264.7 cells for iNOS was performed with a 30-min, one-step solution containing a primary conjugated antibody (iNOS Monoclonal AlexaFluor488, 1:200, Invitrogen) and a nuclear stain (NucSpot Live 650, 1:400, Biotium). All samples were washed and stored, covered by aluminum foil, in flow buffer until flow cytometry analysis.

Flow cytometry was performed on a NovoCyte Quanteon Flow Cytometer System (4 Lasers, Agilent). Gating was determined by analyzing control samples prepared from cells on plain glass, stained for nuclei (NucSpot 650) and actin (AlexaFluor 488 Phalloidin). Nuclear stains were utilized to confirm the location of the single cell population on the forward scatter (FSC) versus side scatter (SSC) plot. The FSC versus nuclear stain fluorescence plots produced defined regions indicating the single cell population, eliminating debris and large clusters. These regions reliably correlated to the expected region on the FSC versus SSC plots. For NHDFs, this relationship was also related to actin fluorescence. In all flow cytometry experiments reported, the nuclear stain was included for RAW 264.7 cells and the actin stain was included for NHDFs as an additional mechanism to confirm selection of the proper cell population. Flow cytometry results were collected as the median value of the fluorescence for a minimum of 1000 cells for NHDF studies and 5000 cells for RAW 264.7 studies. Results are displayed normalized to the control (glass) average, with representative plots of the fluorescence versus count.

#### 2.3.4 Cell phenotype controls

Positive controls were performed for NHDF and RAW 264.7 cells according to existing procedures [[42], [43], [44]]. All cells were cultured in 24-well plates as described. After 24 h of attachment in culture media modified to 5% FBS (for NHDF) and at the standard 10% FBS (for RAW 264.7), cells were exposed to their respective phenotype-inducing cocktails. Activated (+αSMA) NHDFs were induced by the addition of 15 ng/mL TGF-β1 (R&D Systems) for 24 h. Inflammation-positive (+iNOS) RAW 264.7 cells were induced with 25 ng/mL LPS (ThermoFisher) and 10 ng/mL IFN-γ (SinoBiological) for 24 h. Cells were then processed for flow cytometry according to their respective protocols (see section *cell retrieval & staining for flow cytometry*).

#### 2.3.5 Conditioned media study

The media of samples containing RAW 264.7 cells in situ, described above, was saved after the initial 48 h (Fig. 2, Step 2b). At that point, the retrieved media is considered conditioned by the RAW 264.7 cells. Media samples were centrifuged at 400g for 8 min to remove any unattached cells or significant debris. NHDFs were plated at 100,000 cells per well (glass bottom) and incubated for 24 h to allow complete attachment. The media of each well was then removed and replaced by one of the conditioned media samples. These samples were incubated for 48 h. Cells were lifted via trypsinization and subjected to the NHDF αSMA staining procedure and flow cytometry previously described.

### 2.4 Microscopy & analysis

#### 2.4.1 Confocal microscopy

Glass bottom, 24-well plates were used for all imaged samples (#1.5H cover glass, Cellvis). Fluorescent images were obtained via confocal microscopy on an Olympus Fluoview 3000 using UPLXAPO-4X, UPLXAPO-10X, and UPLXAPO-20X objective lenses. The imaging window was the x-y plane. Step sizes for z-stacks were determined in accordance with the native optimization available in the microscope-associated software. Stacks were taken at least 50 μm deep into the substrates. Live cell images were acquired at 37 °C and 5% CO2 utilizing an on-stage incubator (Tokai Hit).

#### 2.4.2 Image post-processing for cell characteristics & pore size

Olympus (.oir) files were opened in FIJI with the Bio-Formats importer [45]. Cells for viability were counted utilizing the Find Maxima function and subsequent manual review. Cell shapes were determined by first projecting the image volume onto a single x-y plane (via Z Project with maximum intensity in FIJI). Then, the actin fluorescence channel was binarized, the cell was outlined, and the shape was filled in to achieve a single connected object. These files were analyzed with the RegionProps function in MATLAB (R2024b, The MathWorks Inc.) to obtain circularity and area. Substrate-only z-stacks were resliced in z to ensure cubic voxels. Images were binarized, despeckled, and outliers were removed (bright and dark, <5 μm). Final z-stacks were imported to a custom Anaconda Online script (Anaconda Software Distribution, ver. 24.9.2) which uses PoreSpy analysis software to obtain the pore size distribution [46].

### 2.5 Materials characterization

All samples for characterization were washed in Milli-Q water and dried. Substrate visualization was performed via scanning electron microscopy (SEM). Samples were sputter coated with 6 nm of iridium to prevent charging. Micrographs were acquired on an FEI Magellan 400 XHR SEM with an excitation voltage of 10 kV at 6 mm working distance. An Oxford energy dispersive x-ray spectroscopy (EDS) attachment (silicon drift detector) was used for chemical analysis. Aztec software was used to identify peaks and calculate weight percents. Fourier Transform Infrared Spectroscopy (FTIR) was performed on a Thermo Scientific Nicolet iS5 with iD5 attenuated total reflectance (ATR) attachment.

### 2.6 Statistical methods

All statistical tests were performed in Microsoft Excel using the Data Analysis ToolPak or in OriginPro (OriginLab), Version 2025. Differences were considered statistically significant when p < 0.05. Cell phenotype control studies, cell viability, and fibroblast phenotype after substrate interaction were compared using two sample t-tests, with the assumption of equal or unequal variance as defined by the F-test of equality of variances. The remaining comparisons were made with the Mann-Whitney *U* test after determining that one or more samples in the set were not normally distributed (Shapiro-Wilk test, p < 0.05). Cell depth distributions were compared using a two sample Kolmogorov-Smirnov test. Variance, N, and p information for each relevant experiment is available in the respective figure captions. Chemical analysis via EDS is presented as mean ± standard deviation.

## 3 Results

### 3.1 Substrate characterization

Representative SEM micrographs of a BTM and a PTM are shown in Fig. 1, which visually demonstrate the pore geometries of these two materials. The BTM has a non-constricting pore network with saddle-like (negative Gaussian) curvatures at its internal surfaces (Fig. 1A), while the spherical cavities of the PTM are connected by much smaller pore throats (1/3 sphere diameter) (Fig. 1B). The pore size distributions of representative BTM and PTM samples were determined via confocal z-stack analysis and show equivalent means and modes, with slight differences in the shape of the distribution, which is expected given the different pore geometries (Fig. S1A). The accuracy of this analysis is limited by the non-uniform fluorescence of the substrates and a loss of consistent z-resolution over large (>80 μm) distances but approximates a useful confirmation of the expected pore sizes.

Chemical analysis of the substrates was performed via EDS and FTIR-ATR. The BTM and PTM are made from the same polymer and subjected to the same post-processing steps, with the exception of HF etching to remove silica particles from the BTM. FTIR-ATR analysis (Fig. S1B) showed expected peaks in the C-H, C-O, C=C, C=O, and O-H regions for both the BTM and PTM spectra. EDS analysis provides a semi-quantitative look at the compositions of the two substrates and is particularly useful in determining the presence or lack thereof of silicon, which is present in the neutrally wetting particles used in the bijel formation step. EDS of the porous samples is limited by the non-uniform surface of the substrates, which is known to affect the accuracy of the measurements [47]. Spots across three different samples per substrate were analyzed, resulting in the following compositions, reported as average weight percents ± standard deviation: BTM: C = 66.1 ± 0.7%, O = 33.9 ± 0.7%, Na = 0.1 ± 0.1%, S = 0.0 ± 0.0% and PTM: C = 68.3 ± 1.1%, O = 31.3 ± 0.9%, Na = 0.2 ± 0.2%, S = 0.2 ± 0.1%. The lack of any silicon signal suggests successful removal of the silica nanoparticles from the BTMs, which is the only discrepancy between how the two substrates are prepared. The elements present (C, O, Na, S) are expected and the difference of <3 wt% between samples is well within the expected variability range for non-flat substrates [48]. The main PEGDA polymer is comprised entirely of carbon and oxygen, while the Rhodamine B degradation agent used on both samples contains both sodium and sulfur. The results suggest a small amount of this agent remains on the substrates after washing. These small quantities of sodium and sulfur are not meaningfully different between the two substrates and are not otherwise expected to notably influence cell behavior, as established by the previously described viability study and the presence of much higher concentrations of both elements in traditional cell culture media [49].

Confocal microscopy was used to analyze the effect of the substrates on cell viability. Transwell studies, which identify any differences caused by the presence of the substrates in the cell culture media, revealed over 80% viability across all control and substrate wells with no statistically significant difference between any two groups (N = 3, p > 0.05). This result demonstrates that there is no significant leaching of molecules which would impact cell behavior. Cell viability within the substrate is visualized via maximum intensity z-projections of live stained cells (Fig. S2), confirming that both cell types survive in both substrates.

### 3.2 Macrophage behavior in situ

**Fig. 3:**
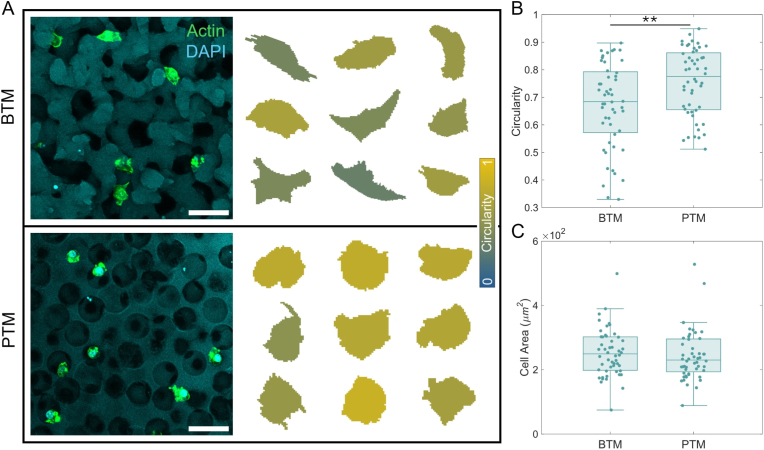
Visualization and analysis of RAW 264.7 cells within porous substrates. (A) The maximum intensity projections for z-stacks of cell images are displayed (left) alongside enlarged and representative cell shapes found in each substrate (right). The cells are color coded by circularity. (B) Quantification of cell circularity within each substrate results in a statistically significant result (N = 52, ∗∗p < 0.01), whereas (C) quantification of cell area shows no significant difference between BTMs and PTMs. Scale bar, 50 μm. (For interpretation of the references to color in this figure legend, the reader is referred to the Web version of this article.)

Representative images of RAW 264.7 cells within a BTM and PTM are shown in Fig. 3. Confocal microscopy images are provided as maximum intensity z-projections with a 50 μm scale bar (Fig. 3A, left). Depth of the original image stacks were 135 μm (PTM) and 80 μm (BTM). 2D analysis of cells in this 3D porous architecture is considered appropriate because both the BTM and the PTM are inherently isotropic. Thus, any variations resulting from the directionality of an individual cell should not differentially accumulate when comparing the two substrates. Cells for shape analysis were conditionally included on the basis of (1) interacting solely with the substrate pore structure and not the flat, cut surface and (2) without significant contact with other cells, which would convolute cell shape bounding. Enhanced visualization of typical cell shapes, recolored according to their circularity, are presented alongside the micrographs (Fig. 3A, right). Macrophages interacting with PTM pore structure appear rounder than those interacting with the BTM. That observation is supported by quantitative circularity analysis of the cells, where circularity is proportional to the ratio of the cell shape area to perimeter squared (Fig. 3B), where a perfect circle has a circularity of 1. While there is a large range of circularities present for cells in both substrate types, cells in the BTM are more likely to reach very low (increasingly non-circular) values and have a statistically significant (N = 52, ∗∗p < 0.01) lower average value. The average areas of the cells on the BTM versus the PTM were not found to be significantly different (p > 0.5) (Fig. 3C).

**Fig. 4:**
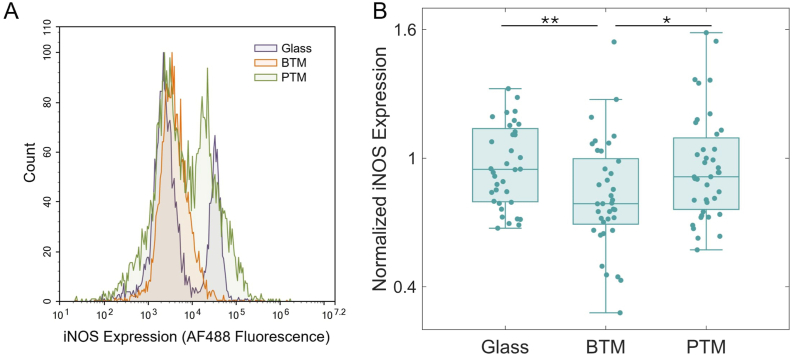
Inflammatory marker (iNOS) expression of RAW 264.7 cells. (A) A representative histogram showing the full fluorescence distribution of cells from each sample. (B) The complete iNOS expression data comparing cells on glass (N = 36), in a BTM (N = 37), or in a PTM (N = 39). The lower overall expression of iNOS in BTM-associated cells was statistically significant in comparison to glass (∗∗p < 0.01) and the PTM (∗p < 0.05). Median expression values are normalized by the mean for cells on glass.

Phenotype of RAW 264.7 cells was determined by staining with pro-inflammatory (M1) marker iNOS and quantifying the relative expression via flow cytometry [29,50]. A control experiment comparing the iNOS expression of uninduced and M1-induced cells was performed (Fig. S3). A representative example of the individual fluorescence versus count distributions for the BTM, PTM, and glass conditions are shown in Fig. 4A. Quantitative results are displayed as the normalized median fluorescence value for each sample (Fig. 4B). The iNOS expression of cells on the BTM (N = 37) was statistically significant in comparison to both glass (N = 36) (∗∗p < 0.01) and the PTM (N = 39) (∗p < 0.05). No difference was found between the glass and the PTM (p > 0.05).

### 3.3 Fibroblast behavior in situ

**Fig. 5:**
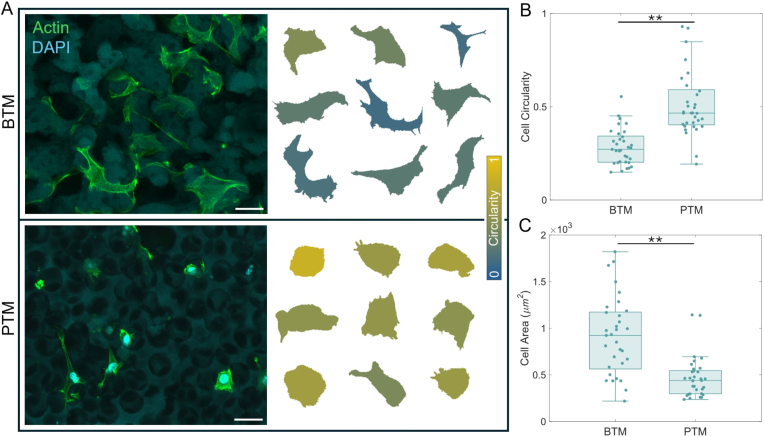
Visualization and analysis of NHDF cells within porous substrates. (A) The maximum intensity projections for z-stacks of cell images are displayed (left) alongside enlarged and representative cell shapes found in each substrate (right). The cells are color coded by circularity. (B) Quantification of cell circularity within each substrate results in a statistically significant result, where the average BTM cell circularity is comparatively lower (N = 33, ∗∗p < 0.01). (C) Quantification of cell area also shows a statistically significant difference (∗∗p < 0.01). Scale bar, 35 μm. (For interpretation of the references to color in this figure legend, the reader is referred to the Web version of this article.)

Representative images of NHDFs within a BTM and PTM are shown in Fig. 5. Confocal microscopy images are provided as maximum intensity z-projections with a 35 μm scale bar (Fig. 5A, left). Similar to macrophages, cells for shape analysis were conditionally included on the basis of (1) only interacting with the pore structure and not the cut surface of the sample and (2) without significant overlap with adjacent cells. Visualization of representative cell shapes, colored according to their circularity, are provided alongside the original micrographs (Fig. 5A, right). Quantitative circularity (Fig. 5B) and cell area (Fig. 5C) analyses showed statistically significant differences between the BTM and PTM (N = 33, ∗∗p < 0.01). NHDFs in BTMs tend to have lower circularities and larger cell areas than those in PTMs, in contrast to RAW 264.7 cells which have lower circularities than those in PTMs but do not show a difference in cell area.

**Fig. 6:**
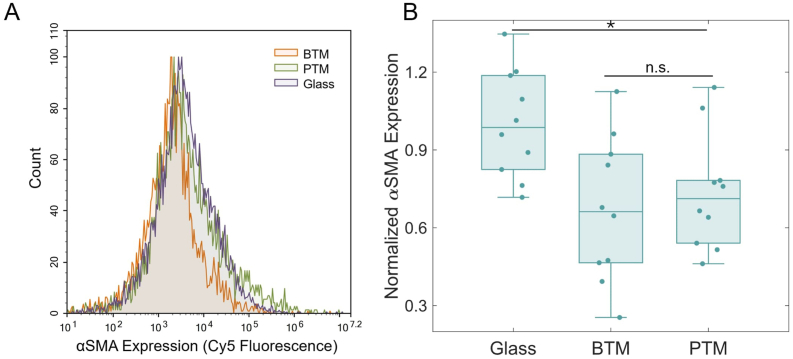
Pro-fibrotic marker (αSMA) expression of NHDF cells. (A) A representative graph showing the full fluorescence distribution of cells from each sample. (B) The complete αSMA expression data comparing cells on glass, in a BTM, or in a PTM. The lower overall expression of αSMA in BTMs and PTMs was statistically significant in comparison to glass (∗p < 0.05), but there was no statistical significance (n.s.) between the BTM and PTM (N = 10). Values are displayed as the sample median normalized by the mean for cells on glass.

Phenotype of NHDF cells was determined by staining with αSMA, a marker of myofibroblast differentiation, and quantifying via flow cytometry [33,35,51]. Myofibroblast differentiation is driven by inflammatory mediators and αSMA-positive myofibroblasts are themselves producers of proinflammatory cytokines and key effectors of fibrotic encapsulation in the foreign body response [52,53]. A control experiment comparing the αSMA expression of uninduced and TGF-β1-induced cells was performed (Fig. S4). Representative examples of the individual fluorescence versus count distributions for each sample type are displayed alongside the full quantitative results for all samples (Fig. 6). The αSMA expression of cells on the BTM versus PTM was not statistically significant (p > 0.05), while compared to cells on glass both the BTM and PTM (∗p < 0.05) were significantly less pro-fibrotic (N = 10 by *t*-test with equal variance).

**Fig. 7:**
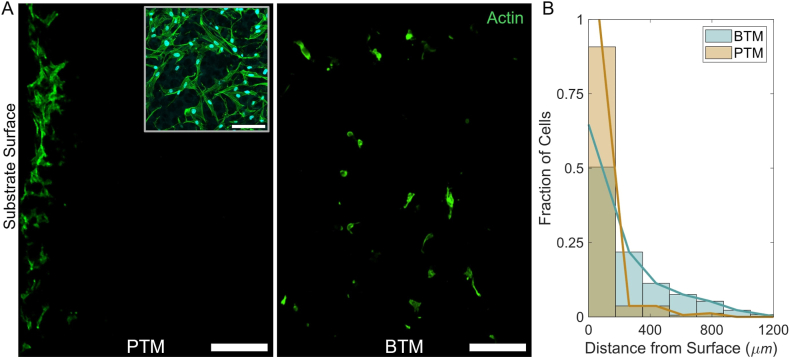
Confocal microscopy of the substrates' cross-sectional areas. (A) Cross sections show actin fluorescence and are displayed with the substrate surface at the leftmost side of the image. The majority of cells in the PTM (left) appear concentrated at the first layers of the substrate, while cells in the BTM distributed more uniformly throughout the substrate volume. The PTM inset image shows a top-down view in a region with high local cell density. (B) Quantification of cell depth within the substrates by histogram with associated linear interpolation (BTM, N = 133; PTM, N = 163). The differences in the distributions are confirmed to be statistically significant via two sample Kolmogorov-Smirnov test (D = 0.448, p < 0.001). Scale bar (A), 200 μm; Scale bar (inset), 100 μm.

We qualitatively observed during cell shape analysis that NHDFs in PTMs were excluded on the basis of interacting with the cut surface of the sample much more frequently than in any other cell-substrate pairing. To further investigate this phenomenon, confocal images of sample cross sections were taken (Fig. 7A). Cells in the PTM appear concentrated in the initial layers of the substrates, while cell distribution is more uniform in depth within the BTM. Quantification of cell depth in both substrates is consistent with this visual analysis, with the two distributions being significantly different (two sample Kolmogorov-Smirnov test, D = 0.448, p < 0.001; Fig. 7B). The inset of Fig. 7A shows a locally dense group of cells on the PTM surface (top-down), which spread across the cut surface of the substrate without meaningfully interacting with the pores. This phenomenon is consistent with our own computational predictions, which show that a cell would not readily traverse through the interconnected throat between two spherical pores [25].

### 3.4 Macrophage-fibroblast influence via conditioned media

**Fig. 8:**
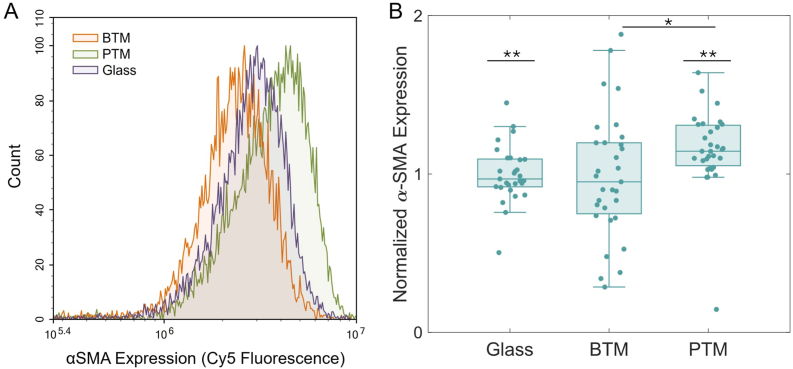
Pro-fibrotic marker (αSMA) expression of NHDF cells after exposure to RAW264.7–BTM or –PTM conditioned media. (A) A representative graph showing the full fluorescence distribution of cells from each sample. (B) The complete αSMA expression data comparing cells exposed to conditioned media from RAW 264.7 cells on glass (N = 29), in a BTM (N = 31), or in a PTM (N = 31). The difference in expression between the BTM-associated and PTM-associated samples is statistically significant (∗p < 0.05), and between the glass and PTM (∗∗p < 0.01).

NHDFs plated on glass were exposed to conditioned media reserved from samples of RAW 264.7 cells on glass, BTMs, and PTMs (Fig. 2). Those NHDFs were then analyzed for pro-fibrotic character via αSMA (Fig. 8). NHDFs in BTM-associated (N = 31) wells exhibited lower αSMA expression than in PTM-associated (N = 31) wells (∗p < 0.05). PTM-associated NHDFs also exhibited higher αSMA expression than glass-associated (N = 29) NHDFs (∗∗p < 0.01).

## 4 Discussion

Fibroblasts and macrophages seeded in BTMs and PTMs were examined after 48 h. For the first time, the effect of BTM microstructure on immune cell shape and phenotype was studied in vitro. The BTMs were analyzed alongside particle-templated materials (PTMs), which were synthesized and shown to be chemically equivalent, but morphologically different. Macrophages on BTMs exhibited a combination of lower cell circularity and lessened inflammation, in comparison to the more circular and more inflamed cells interacting with the PTMs (Fig. 3, Fig. 4). Our results suggest a direct relationship between the BTM's unique pore architecture, induced cell shape, and macrophage phenotype. This proposed relationship is supported by existing research which shows connections between macrophage elongation and pro-healing phenotype [26], as well as between substrate curvature and actin organization [14]. In addition, the distinct pore geometries and local surface curvatures of the BTM and PTM are expected to impose different cell-substrate contact patterns, producing different intracellular stress states. Recent work has demonstrated that the spatial organization of substrate contact cues governs intracellular mechanical tension and downstream cell behavior [54,55], providing a mechanistic framework through which architectural differences alone could drive the morphological and phenotypic effects observed here. Furthermore, such distinct environments may have implications beyond acute phenotypic response; recent evidence shows that intracellular mechanical state can regulate cellular aging and associated inflammation in a dose-dependent manner [56], suggesting that the chronic mechanical inputs imposed by pore architecture could influence longer-term cellular programs relevant to the foreign body response.

In contrast, fibroblasts seeded into BTMs were both larger and less circular than PTM-seeded fibroblasts but did not have different pro-fibrotic outcomes (Fig. 5, Fig. 6). Both cell types exhibited higher circularity when interacting with the PTM pore structure, but only fibroblasts showed a difference in cell area. This discrepancy suggests that there is another factor at play. Many fibroblasts interacting with the PTM were observed to preferentially spread along the cut, flat surface of the substrate, crossing over top of multiple pores (Fig. 7A, inset). Subsequently, only a small fraction of fibroblasts interacted solely with the PTM pore structure. The NHDF-PTM combination was the only cell-substrate pair where this surface-preferential cell adhesion was observed. We conclude that a combination of fibroblast size (notably larger than macrophages (Figs. 3C and 5C)) and the constricting interconnect regions of the PTMs resulted in a size exclusion effect which dominated the NHDF-PTM behaviors. Essentially, the NHDFs interacted with the PTM as if it were a largely planar structure, nearly unaffected by or unaware of the porous regions beyond the surface. Cross-sectional cell depth analysis further supports this theory (Fig. 7). Fibroblasts seeded on PTMs tended to collect at the injection surface of the substrate, in contrast to cells seeded on BTMs, which spread throughout the porous channels of the substrate. Given that the pro-fibrotic metric used is for the total population of substrate-adhered cells, the result of the NHDF-PTM combination does not address how the PTM pore structure itself affects NHDF fibrotic state. If a significant portion of the measured cells avoid entering the pore structure altogether, then a true comparison of cell behavior within the BTM and PTM microstructures cannot be performed for fibroblasts at this pore size. Additional methods could be used to allow the study of this relationship, such as a prescribed chemotaxis gradient which would force the NHDFs to interrogate the PTM architecture. However, this work seeks to understand how the BTM and PTM structures, without convoluting factors, affect cell behavior. Therefore, we instead investigate how the effects of these substrates on macrophage behavior may cause downstream effects via cell-cell signaling.

A conditioned media test was performed to determine the effect, if any, of introducing macrophage-conditioned media to fibroblasts grown on glass (Fig. 2). This experiment exposed fibroblasts to the factors excreted by macrophages within the substrates by direct transplantation of the chemical environment. Doing so revealed that not only can BTMs induce phenotype changes to macrophages directly, but also that the induced changes are significant enough to push fibroblasts into a lower pro-fibrotic state without any direct fibroblast-BTM contact (Fig. 8). While this establishes a functional link between macrophage-substrate interaction and downstream fibroblast phenotype, the specific mediators responsible have not been identified. Blocking experiments targeting candidate cytokines and signaling pathways represent a critical next step in validating the causal chain. Overall, these findings suggest that substrate architecture influences downstream cell phenotypes through its initial effects on macrophage polarization, pointing to the broader cascade in which material-driven changes in one cell type propagate through the local signaling environment to affect others.

Our study provides the first look at how BTM and PTM topographies alone can influence the behavior of specific immune cells, and how that resultant behavior can cause downstream effects through paracrine signaling. The relatively low cell density used in these studies was designed to minimize physical cell-cell interactions and to match the single-cell conditions of prior computational predictions [25]. At higher cell densities, macrophage functional phenotype is known to be further modulated by density-dependent signaling [57,58]. Understanding how individual cell-substrate responses identified here are integrated into collective behavior at physiological densities represents an important direction for future work. We note that the present study focused on M1 polarization. While we observed a significant reduction in inflammatory expression in BTM-associated macrophages, the inclusion of M2-associated markers would provide a more comprehensive characterization of macrophage polarization within these substrates. Future studies incorporating a panel of both M1 and M2 markers are warranted to fully elucidate the polarization landscape within BTMs and PTMs. The results of this study motivate exploration of the interaction between these substrates and additional immune cell types involved in the foreign body response, including neutrophils and T cells, as well as vascular endothelial cells, which are critical to the vascularization observed in previous in vivo work [9]. Specific investigation into the signaling pathways responsible for the established relationship between pore morphology, cell shape, and immune response in these substrates could provide new insight into best practices for designing new biomaterials with superior cell control.

## 5 Conclusions

We report the investigation of immune cell response to the morphological properties of a new class of porous biomaterials. Our substrates, templated from bijels, have continuous pore networks with uniform pore size and negative Gaussian surfaces. Fibroblasts and macrophages were seeded into BTMs and analyzed after 48 h. The pore structure of the BTM caused seeded macrophages to be less circular and less inflamed than a chemically equivalent material with a different pore architecture. This anti-inflammatory effect on macrophages produced conditioned media which alone was able to cause a reduction in pro-fibrotic activation in fibroblasts. These results indicate that there is a direct relationship between pore structure and macrophage polarization, mediated by the influence on cell shape, and introduce BTMs as a unique platform to modulate macrophage behavior. Future studies should seek to elucidate the effects of these materials on the other implicated cell types discussed and to discern how those effects interplay with one another. A breadth of understanding in this area would greatly inform the use of biomaterial structural elements in governing cell behavior.

## Funding

The authors acknowledge the use of facilities and instrumentation at the UC 10.13039/100016829Irvine Materials Research Institute (10.13039/100016829IMRI), which is supported in part by the 10.13039/100000001National Science Foundation through the UC 10.13039/100008476Irvine 10.13039/100013111Materials Research Science and Engineering Center (DMR-2011967). SEM and 10.13039/100004679EDS work was performed using instrumentation funded in part by the 10.13039/100000001National Science Foundation 10.13039/100016511Center for Chemistry at the Space-Time Limit (CHE-0802913). Use of confocal microscope Fluoview3000 (10.13039/100009734Olympus) inside the 10.13039/100006520Edwards Lifesciences Foundation Cardiovascular Innovation and Research Center was supported by (10.13039/100000002NIH 1S10OD025064-01A1). Funding for this study was partly provided by the National Science Foundation (DMS-1953410). The graphical abstract was made using BioRender (Gonthier, A. (2025) https://BioRender.com/a91n288). Fig. 2 was made using BioRender (Gonthier, A. (2025) https://BioRender.com/n40o557).

## CRediT authorship contribution statement

**Alyse R. Gonthier:** Data curation, Formal analysis, Investigation, Methodology, Writing – original draft, Writing – review & editing. **Elliot L. Botvinick:** Conceptualization, Formal analysis, Funding acquisition, Investigation, Supervision, Validation, Writing – review & editing. **Ali Mohraz:** Conceptualization, Funding acquisition, Investigation, Supervision, Validation, Writing – review & editing.

## Declaration of competing interest

The authors declare that they have no known competing financial interests or personal relationships that could have appeared to influence the work reported in this paper.

## Data Availability

Data will be made available on request.
