# Supplementary material for: Uniform pore morphology of bijel-templated materials reduces cell circularity and inflammatory expression of macrophages
Source: Mater Today Bio. 2026 Apr 29;38:103175. doi: 10.1016/j.mtbio.2026.103175 (PMC13147999; doi:10.1016/j.mtbio.2026.103175)
Supplement: Multimedia component 1 [file mmc1.pdf]

Supplementary Information for

Uniform Pore Morphology of Bijel-Templated Materials Reduces Cell Circularity and Inflammatory Expression of Macrophages

Authors

Alyse R. Gonthier[a], Elliot L. Botvinick[bcde*], Ali Mohraz[af*]
Emails: agonthie@uci.edu, ebotvini@uci.edu, mohraz@uci.edu

[a] Department of Materials Science and Engineering, University of California, Irvine, CA 92697, USA
[b] Department of Biomedical Engineering, University of California, Irvine, CA 92697, USA
[c] Department of Surgery, University of California, Irvine, CA 92697, USA
[d] Beckman Laser Institute, University of California, Irvine, CA 92697, USA
[e] Edwards Lifesciences Foundation Cardiovascular Innovation and Research Center, University of California, Irvine, CA 92697, USA
[f] Department of Chemical and Biomolecular Engineering, University of California, Irvine, CA 92697, USA

*Address correspondence to: Ali Mohraz – Email: mohraz@uci.edu and Elliot Botvinick – Email: ebotvini@uci.edu

Supplemental Figures

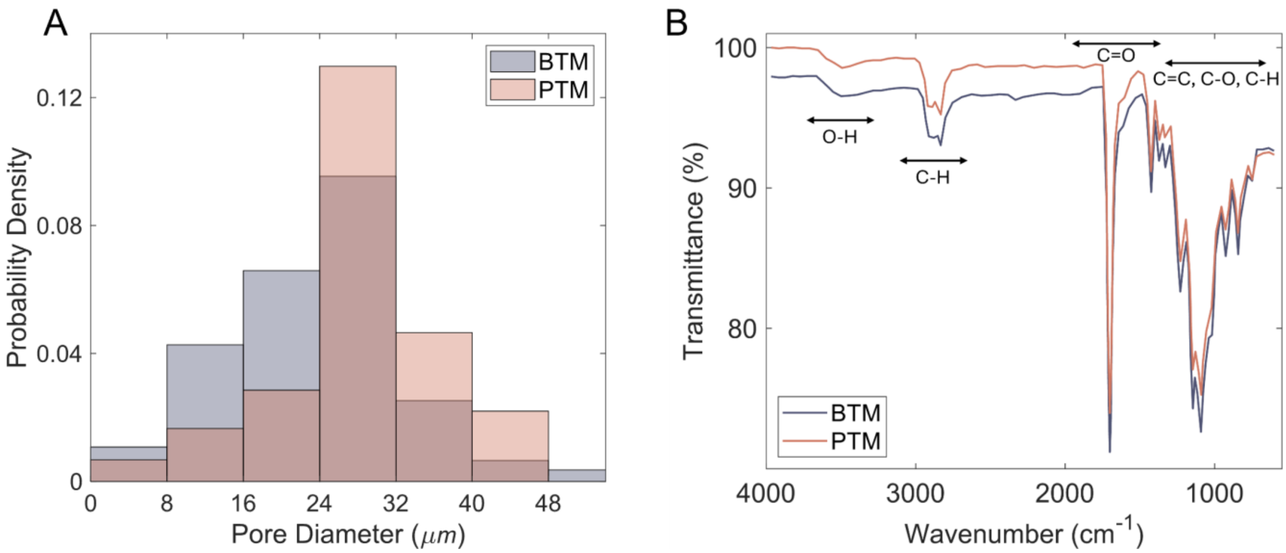

Figure S1. (A) Pore size distributions of representative BTM and PTM substrates obtained via PoreSpy analysis of confocal z-stacks. (B) FTIR-ATR spectra of the two substrates.

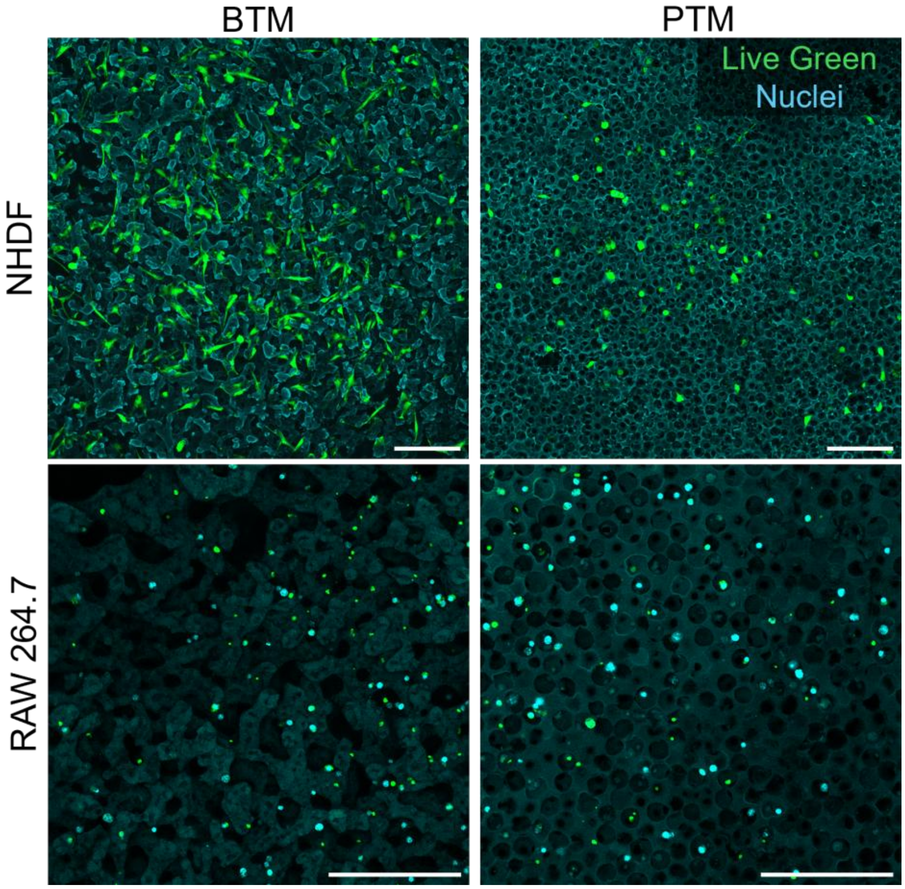

Figure S2. Live fibroblasts and macrophages in situ. Live fibroblasts in the BTM (top left) appear spread and elongated, while fibroblasts in the PTM (top right) appear confined. Live macrophages in both substrates (bottom) are visible, though shape is not discernable with the Live Green stain. Scale bar, 200 μm.

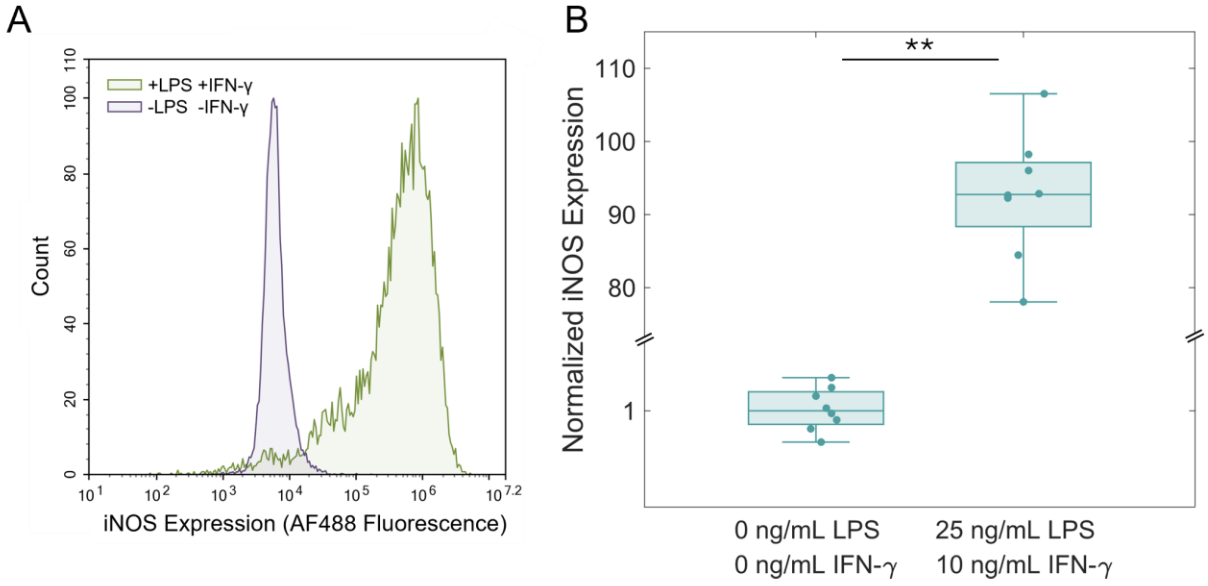

Figure S3. Positive control for inflammatory expression by RAW 264.7 cells. (A) Representative samples showing the distribution of inflammatory fluorescence in cells exposed to a known M1-inducing cocktail compared to uninduced cells. (B) Comparison of inflammatory marker expression shows cells exposed to the M1-inducing cocktail are more inflamed, with statistical significance ($N = 8$, $^{**}p < 0.01$) via t-test with equal variance.

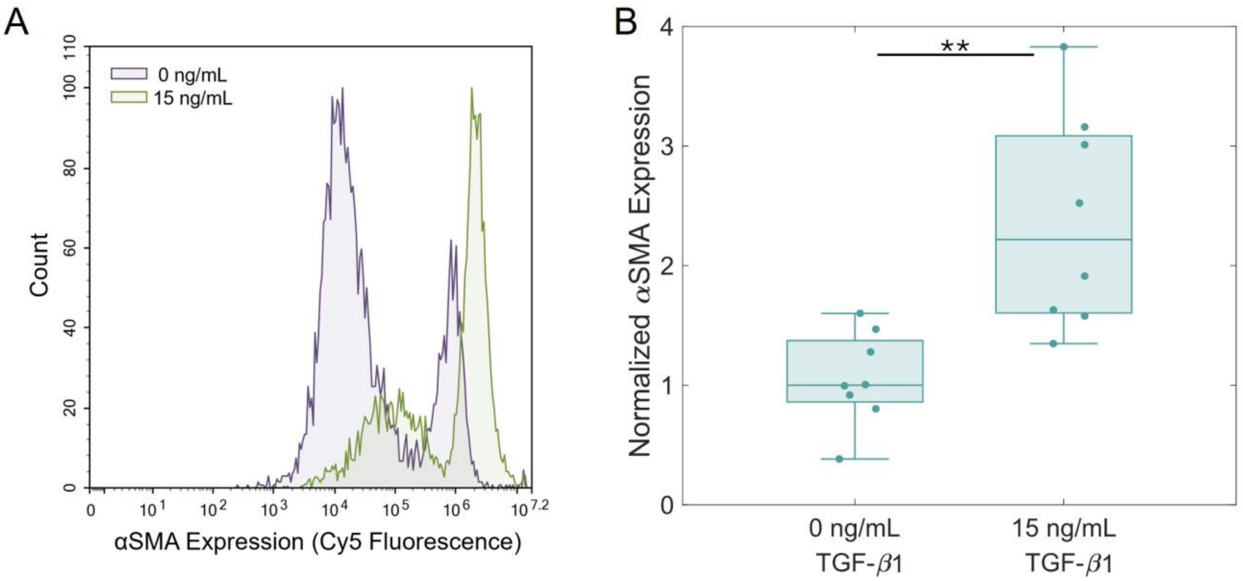

Figure S4. Positive control for expression of the pro-fibrotic marker αSMA in NHDFs. (A) Representative samples showing the distribution of αSMA fluorescence in cells exposed to 0 or 15 ng/mL TGF-β1. (B) Comparison of αSMA expression shows cells exposed to 15 ng/mL TGF-β1 are more fibrotic, with statistical significance ($N = 8$, $^{**}p < 0.01$) via t-test with unequal variance.
